# *In vitro* expansion of Wilms’ tumor protein 1 epitope-specific primary T cells from healthy human peripheral blood mononuclear cells

**DOI:** 10.1016/j.xpro.2023.102053

**Published:** 2023-01-30

**Authors:** Sanne van der Heijden, Donovan Flumens, Maarten Versteven, Stefanie Peeters, Hans De Reu, Diana Campillo-Davo, Yannick Willemen, Benson Ogunjimi, Viggo Van Tendeloo, Zwi N. Berneman, Sébastien Anguille, Evelien Smits, Eva Lion

**Affiliations:** 1 Laboratory of Experimental Hematology (LEH), Vaccine & Infectious Disease Institute (VAXINFECTIO), Faculty of Medicine and Health Sciences, University of Antwerp, Universiteitsplein 1, 2610 Antwerp, Belgium; 2 Center for Oncological Research (CORE), Integrated Personalized & Precision Oncology Network (IPPON), University of Antwerp, Universiteitsplein 1, 2610 Antwerp, Belgium; 3 Division of Hematology, Antwerp University Hospital (UZA), Drie Eikenstraat 655, 2650 Edegem, Belgium; 4 Centre for Health Economics Research & Modeling Infectious Diseases (CHERMID), VAXINFECTIO, University of Antwerp, Universiteitsplein 1, 2610 Antwerp, Belgium

**Keywords:** Cell Biology, Cell Culture, Cell Isolation, Flow Cytometry/Mass Cytometry, Cancer, Health Sciences, Immunology

## Abstract

Wilms’ tumor protein 1 (WT1) is a tumor-associated antigen overexpressed in various cancers. As a self-antigen, negative selection reduces the number of WT1-specific T cell receptors (TCRs). Here, we provide a protocol to generate WT1_37-45_-specific TCRs using healthy human peripheral blood mononuclear cells. We describe the expansion of WT1-specific T cell clones by two consecutive *in vitro* stimulations with autologous WT1_37-45_-pulsed dendritic cells and peripheral blood lymphocytes. We then detail the detection with human leukocyte antigen/WT1_37-45_ tetramers.

## Highlights

• Robust fully autologous *in vitro* expansion of WT1-reactive T cells from healthy donors
• Two consecutive one-week stimulations with autologous peptide-pulsed DCs and PBLs
• Steps for tetramer staining, allowing WT1_37-45_-specific T cell purification
• Expanded T cells can serve for applications analyzing TCR repertoire or isolating TCRs

## Abstract (editor highlights)

Publisher’s note: Undertaking any experimental protocol requires adherence to local institutional guidelines for laboratory safety and ethics.

## Graphical abstract

**Figure:**
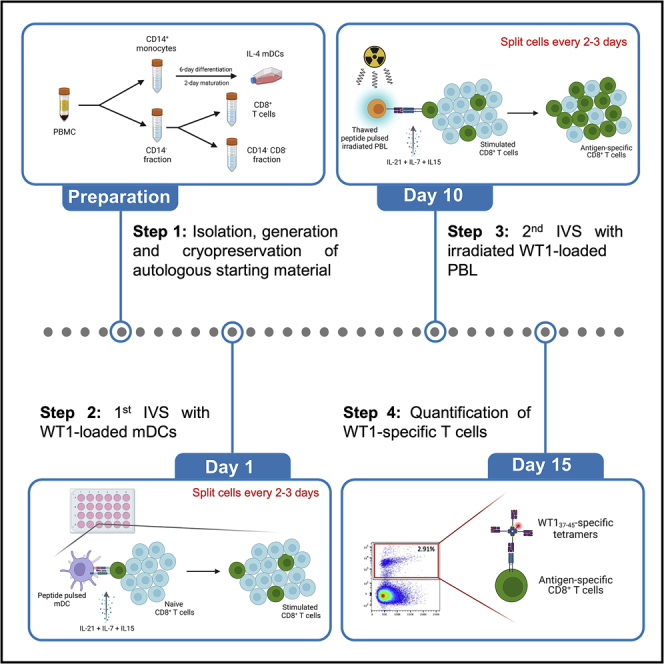

## Teaser

Wilms’ tumor protein 1 (WT1) is a tumor-associated antigen (TAA) overexpressed in various cancers. As a self-antigen, negative selection reduces the number of WT1-specific T cell receptors (TCRs). Here, we provide a protocol to generate WT1_37-45_-specific TCRs using healthy human peripheral blood mononuclear cells. We describe the expansion of WT1-specific T cell clones by two consecutive *in vitro* stimulations with autologous WT1_37-45_-pulsed dendritic cells and peripheral blood lymphocytes. We then detail the detection with human leukocyte antigen/WT1_37-45_ tetramers.

## Before you begin

This protocol describes the specific steps for *in vitro* expansion of WT1 epitope-specific primary CD8^+^ T cells from healthy donor PBMCs. By means of human leukocyte antigen (HLA)-A∗02:01 (hereafter termed as HLA-A2)/WT1 tetramer staining, expanded specific T cells can be analyzed to assess WT1 epitope-specific T-cell reactivity, sequenced for TCR repertoire, or (single-cell) sorted to generate T-cell clones for the discovery of epitope-specific TCR sequences for the development of TCR-engineered cell therapies. Purified CD8^+^ T cells are expanded in two rounds of *in vitro* stimulation (IVS). For the first IVS of 8 days, autologous dendritic cells (DCs) are used, which are differentiated from freshly isolated CD14^+^ monocytes and after maturation pulsed with immunodominant HLA-A2-restricted WT1_37-45_ epitope. After priming of the naïve CD8^+^ T cells, irradiated autologous peptide-pulsed CD14^-^ and CD8^-^ depleted peripheral blood lymphocytes (PBLs) are used for the second IVS of 8 days. Expanded WT1-specific T cells are detected with fluorochrome-labeled WT1-specific tetramers, allowing quantification and sorting of epitope-specific T cells, for further downstream applications.

### Institutional permissions

We highly recommend researchers to have hepatitis B vaccination when working with human blood. Other recommended vaccinations are dependent on the country and research institute. Apply safety precautions, such as the use of biological safety cabinets, gloves, and protective clothing, while handling blood products. Experiments should be performed in accordance with all relevant institutional and governmental ethics regulations, and informed consent should be obtained from all subjects for the experimental use of human blood. This study was approved by the Ethics Committee of the Antwerp University Hospital and the University of Antwerp (reference number 15/19/210).

### Preparation of HLA-A2/WT1_37-45_ tetramers for surface staining

**Timing: 2 h**

The major histocompatibility complex (MHC) tetramers are oligomers consisting of four biotinylated MHC monomers. In this case, each HLA-A2 monomer presents the WT1_37-45_-peptide. By adding streptavidin, which contains four biotin binding sites, four MHC monomers are grouped together to form a tetramer specific for WT1_37-45_-specific TCRs. The streptavidin molecule is linked to a fluorochrome to allow direct staining of the TCR for flow cytometric acquisition. Both the peptide and the MHC heavy chain let the tetramer bind specifically to the TCR of interest. Fluorochrome-labeled tetramers used in this protocol were produced in-house starting from monomers. Alternatively, MHC tetramers can be purchased from a commercial vendor.

**CRITICAL:** Fluorochrome-labeled tetramers are sensitive to light and temperature and therefore must be prepared on ice and in the dark.

1. Tetramerization.
  a. Thaw the MHC monomers and keep them on ice.
  b. Calculate the amount of streptavidin-APC to be added to the MHC monomers.
    ***Note:*** The amount of streptavidin-allophycocyanin (APC) to be added depends on the molarity of the streptavidin. Here, a 4:1 monomer:streptavidin ratio is used.
  c. Add 1/5 of the total amount of streptavidin-APC to the monomers.
  d. Mix for 20 min on ice in the dark using a shaker.
  e. Repeat steps 1c and 1d 4 times.
  f. Store the tetramers protected from light at 4°C for up to 2 months.

### HLA-A2 typing by surface staining

**Timing: 50 min**

The procedures described are performed on HLA-A2^+^ donors. Therefore, as a preparatory step, the expression of surface HLA-A2 in whole blood of healthy donors is to be determined, here by flow cytometry.

2. HLA-A2 staining.
  a. Transfer 100 μL of whole blood into a non-sterile FACS tube.
  b. Add 100 μL of a mouse anti-human HLA-A2 primary antibody and incubate for 15 min at room temperature (20°C–22°C).
  c. To lyse red blood cells (RBC), add 4 mL of RBC lysis buffer and incubate for 10 min at room temperature.
  d. Centrifuge tubes at 480 × *g* for 5 min, 20°C–22°C and decant supernatant.
  e. Add 1 μL fluorescein isothiocyanate (FITC)-conjugated rabbit anti-mouse secondary antibody and incubate for 10 min at room temperature in the dark.
  f. Add 2 mL of FACS buffer and wash by centrifuging at 480 × *g* for 5 min, 20°C–22°C and discard buffer.
  g. Add 100 μL of FACS buffer and determine HLA-A2 status by flow cytometry.

***Note:*** In order to rule out non-specific binding of the secondary antibody, include as a negative control a sample in which staining is performed only with the secondary antibody without adding the primary antibody.

***Note:*** HLA-A2 staining can be performed on either whole blood or isolated PBMCs. When staining PBMCs, the sample does not have to be lysed and can be washed after incubation of the primary antibody with 2 mL of FACS buffer instead.

### Purification of T cells and generation of mature monocyte-derived DCs from healthy PBMCs

**Timing: 8 days**

**Figure 1:**
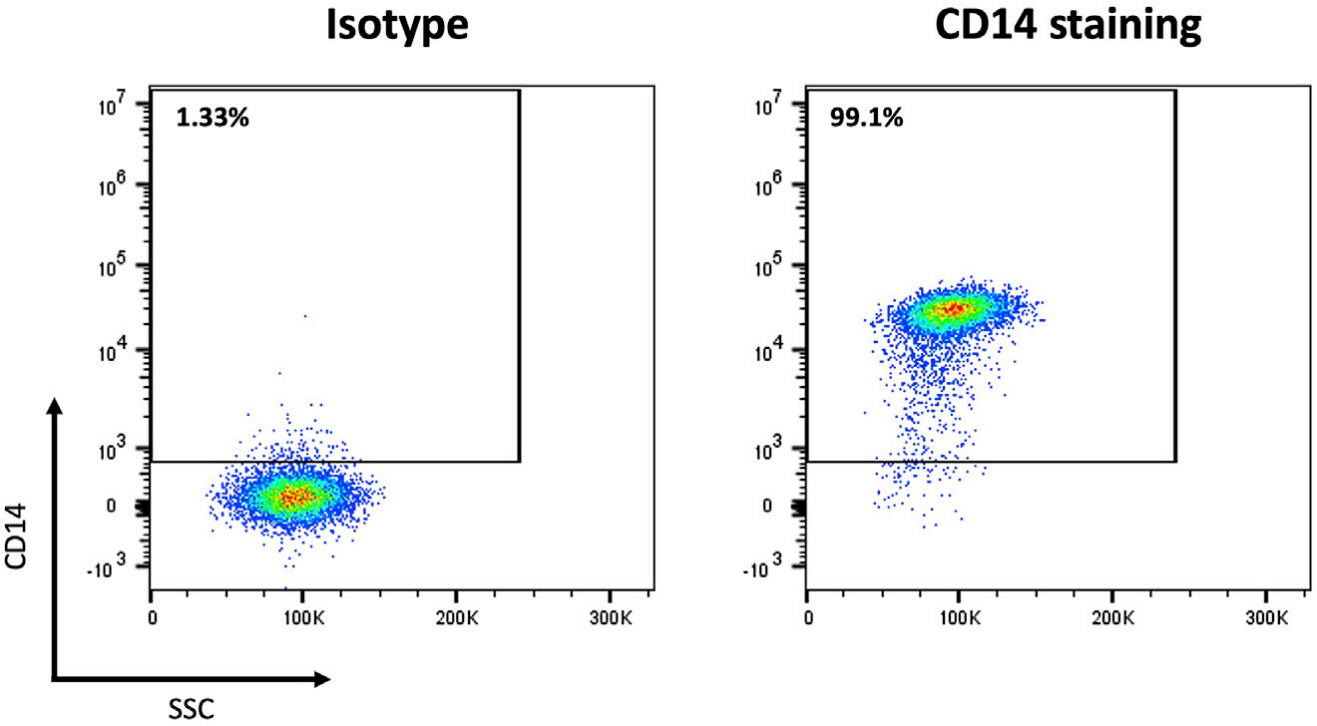
Representative flow cytometric plots of the percentage of CD14^+^ cells after immunomagnetic enrichment from PBMC Gates were set on viable cells gating on the forward scatter (FSC) and side scatter (SSC) pseudocolor plots. An isotype control is used to determine CD14 positivity. A purity between 90%–99% can be expected after immunomagnetic isolation.

In this protocol, freshly isolated PBMCs obtained from buffy coat preparations of healthy individuals that are positive for the HLA-A2 allele are used. The following section describes the steps leading to the isolation and maturation of CD14^+^ monocytes from PBMCs by immunomagnetic cell labeling using anti-CD14 microbeads and subsequent magnetic-activated cell sorting (MACS) from PBMCs. For a standard experiment, it is recommended to start with 0.8–1.0 × 10^9^ PBMC to obtain an average monocyte yield of 0.8–1.0 × 10^8^ cells based on the expected average frequency of monocytes in PBMC (10%–15%). Following CD14^+^ monocyte isolation, the autologous monocyte-depleted CD14^-^ fraction (mainly, PBLs) is subjected to positive anti-human CD8 MACS-isolation to obtain CD8^+^ T cells. The yield of CD8^+^ T cells after isolation may be variable between donors. On average, 7.5 × 10^7^ CD8^+^ T cells are isolated when starting from 6.0 × 10^8^ PBL. Purity of the CD14^+^ (Figure 1) and CD8^+^ (Figure 4) enriched fractions is determined by immunofluorescent staining and flow cytometry. Next, CD14^+^ monocytes are differentiated with interleukin (IL)-4 and granulocyte-macrophage colony-stimulating factor (GM-CSF) and matured to generate mature IL-4 DC which are phenotypically characterized by flow cytometry. Finally, primary CD8^+^ T cells are cryopreserved until further use with autologous mature DCs.

3. CD14^+^ monocyte isolation from fresh (not-cryopreserved) PBMCs.
  ***Note:*** For human CD14^+^ monocyte positive selection, please refer to the manufacturer’s instructions: https://www.miltenyibiotec.com/upload/assets/IM0001260.PDF). The following CD14^+^ monocyte isolation protocol is used, based on these instructions.
  a. Magnetic labeling and separation of CD14^+^ monocytes.
    i. Count PBMCs and collect the amount of PBMCs needed for isolating of the anticipated amount of CD14^+^ monocytes.
      ***Note:*** Use at most 1.0 × 10^9^ PBMC, and at most 1.0 × 10^8^ positively selected cells per LS magnetic column, according to the product specifications.
    ii. Wash PBMCs with MACS buffer, centrifuge at 480 × *g* for 5 min, 20°C–22°C and decant MACS buffer.
    iii. Add 8 μL of MACS buffer per 1.0 × 10^6^ PBMCs.
    iv. Add 2 μL of CD14 microbeads per 1.0 × 10^6^ PBMC and mix well.
    v. Incubate for 15 min at 2°C–8°C.
    vi. Add MACS buffer to fill the tube to 50 mL (or 10 times the initial volume).
    vii. Centrifuge at 480 × *g* for 5 min and decant MACS buffer.
    viii. Resuspend cell pellet in 5 μL of MACS buffer per 1.0 × 10^6^ total cells.
    ix. Place LS columns in the magnetic field of a suitable MACS Separator.
    x. Prepare column by rinsing with 3 mL of MACS buffer. Wait until the buffer completely passes through the column reservoir.
    xi. Apply cell suspension onto the column and collect the CD14^-^ cell fraction that passes through in a 50 mL Falcon tube.
    xii. Wash the column 3 times with 3 mL of MACS buffer. Only add new buffer when the column reservoir is completely empty and avoid air bubbles.
    xiii. Remove column from the separator and place it on a 15 mL Falcon tube.
    xiv. Add 5 mL of MACS buffer onto the column outside the magnetic field and immediately flush out the magnetically labeled cells (representing the positive cell fraction; CD14^+^ monocytes) by firmly pushing the plunger into the column.
    xv. Add MACS buffer up to 10 mL to the tube of CD14^+^ cells.
    xvi. Count the CD14^+^ and CD14^-^ cell fractions and determine viability.
      ***Note:*** One could use an automatic cell counter (e.g., Horiba ABX Micros 60 (Horiba, Ltd.)) and propidium iodide (PI) staining to determine the cell count and viability flow cytometrically, respectively.
      ***Note:*** Isolate autologous naïve CD8^+^ T cells from the CD14^-^ cell fraction (*vide step 7*). If the CD8^+^ T cells are isolated on the same day, add MACS buffer up to 50 mL to the tube of CD14^-^ cells. If CD8^+^ T cells are isolated the next day, resuspend in 30 mL of AIM-V medium and place on a roller overnight (15–18 h).
    xvii. Centrifuge CD14^+^ selected cells at 480 × *g* for 5 min.
    xviii. Decant buffer and resuspend CD14^+^ monocytes in DC culture medium at a seeding density of at least 1.3 × 10^6^ cells/mL.
  b. Check monocyte cell purity by flow cytometry in the CD14^+^ selected cell fraction.
    i. Take two times 0.1 × 10^6^ cells and transfer into non-sterile FACS tubes: use one tube to stain the cells by adding 1 μL IgG2b FITC as isotype control (adjust the isotype and the amount added if required) and use the second to stain CD14^+^ isolated monocytes by adding 1 μL CD14-FITC.
    ii. Incubate 15 min at room temperature (20°C–22°C) in the dark.
    iii. Wash once with 2 mL of FACS buffer. Centrifuge at 480 × *g* for 5 min, 20°C–22°C and discard supernatant.
    iv. Add 100 μL of FACS buffer and analyze by flow cytometry.
      ***Note:*** Purity is expected to be >95% (monocytes among live cells) (Figure 1). If viability is <90%, take it into account for monocyte differentiation seeding density (see below).

4. CD14^+^ monocyte differentiation and maturation into DCs.
  a. Resuspend isolated CD14^+^ monocytes at 1.3 × 10^6^ cells/mL in DC culture medium supplemented with 20 ng/mL of IL-4 and 800 IU/mL of GM-CSF.
  b. Transfer to a flat-bottomed culture recipient (e.g., T175 flask or 6-well plates).
  c. Place horizontally in the incubator for 6 days at 37°C, 5% CO2.
  d. At day 6, add maturation cocktail, consisting of 20 ng/mL of tumor necrosis factor alfa (TNF-α) and 1 μg/mL of prostaglandin E2 (PGE_2_) to the flask.
  e. Incubate cells for an additional 40–44 h at 37°C.

***Note:*** These conditions were previously shown to result in mature IL-4 DCs.^1^

**Figure 2:**
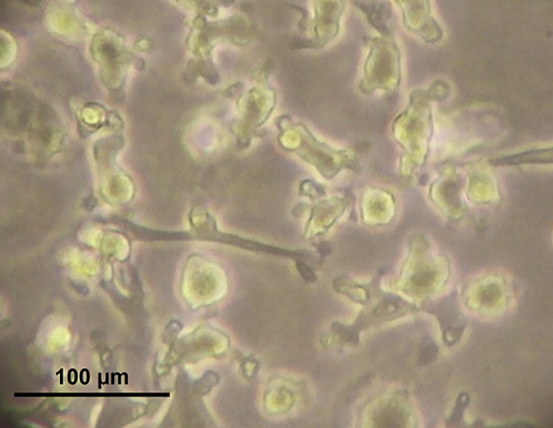
Microscopic image of mature IL-4 DCs Image of cultured monocyte-derived mature IL-4 DCs was taken at a 400× magnification. Scale bar, 100 μm.
  b. Gently tap on the back and sides of the flask for DCs to detach (alternatively, use a cell scraper or Pasteur pipette when using a 6-well plate).
  c. Collect the cells in a 50 mL Falcon tube.
  d. Wash the flask (or wells) once by adding of 25 mL (or 4 mL) cold phosphate-buffered saline (PBS)-ethylenediaminetetraacetic acid (EDTA) buffer to harvest the remaining cells.
  e. Incubate for 10 min at 4°C and transfer the remaining cells to the 50 mL Falcon tube.
  f. Check the flask under the microscope to ensure that all cells have been harvested.
    **CRITICAL:** If many cells are remaining in the flask, repeat the washing step with cold PBS-EDTA buffer.
  g. Centrifuge the tubes at 480 × *g* for 5 min, 20°C–22°C and discard buffer.
  h. Resuspend cell pellet in 10 mL of serum-free Roswell Park Memorial Institute (RPMI) medium and count the viable cells (problem 1).
    ***Note:*** DC viability is expected to be >90%.

5. Harvest mature IL-4 DCs.
  a. Prior to harvesting, analyze the morphology of the mature IL-4 DCs microscopically. Figure 2 shows a microscopic image of mature IL-4 DCs.
    ***Note:*** Mature IL-4 DCs grow in a monolayer and are characterized by long and strongly branched dendrites, indicating maturation of the cells. Mature IL-4 DCs can adhere firmly to the bottom of the culture recipient.

6. IL-4 DC membrane phenotyping.
  a. Transfer 0.4 × 10^6^ harvested DCs into non-sterile FACS tube.
  b. Centrifuge the tubes at 480 × *g* for 5 min, 20°C–22°C and discard supernatant.
  c. Resuspend the cell pellet in 400 μL of FACS buffer.
  d. Block the sample with 4 μL of mouse gamma globulins and incubate for 10 min at room temperature.
    ***Note:*** Blocking is performed with 1 μL mouse gamma globulin per 100 μL of DC suspension to reduce unwanted antibody binding to Fc receptors.
    **CRITICAL:** Do not wash after incubation.
  e. Transfer 0.1 × 10^6^ blocked cells per sample to four non-sterile FACS tubes for surface staining with the fluorescently labeled antibody panel A and B (Table 1).

**Table 1:** Antibody staining panels for flow cytometric analysis of mature IL-4 DC phenotype on a CytoFlex cytometer

Panel A	FITC	Volume	PE	Volume	PE-Cy7	Volume
Tube 1: CD209/CD14/CD86	CD209	3 μL	CD14	1 μL	CD86	1 μL
Tube 2: Isotype control panel A	IgG2b	1 μL	IgG2b	1 μL	IgG1	1 μL

Panel B	FITC	Volume	PE	Volume	PE-Cy7	Volume
Tube 1: CD83/CD80/CD14	CD83	1 μL	CD80	1 μL	CD14	1 μL
Tube 2: Isotype control panel B	IgG1	1 μL	IgG1	1 μL	IgG1	1 μL
  f. Incubate tubes for 15 min at room temperature in the dark.
    ***Optional:*** In the meantime, prepare a 7-Aminoactinomycin D (7-AAD) solution to add as a viability marker to the staining panels: mix 2 μL of 7-AAD and 50 μL of FACS buffer per sample of the panel.
  g. Add 2 mL of FACS buffer and wash by centrifuging at 480 × *g* for 5 min, 20°C–22°C and carefully discard the buffer.
  h. Add 50 μL of 7-AAD solution per sample and incubate for 10 min at room temperature.
  i. Analyze samples on a flow cytometer. A representative phenotypic profile of mature IL-4 DCs is shown in Figure 3.
    ***Note:*** Mature IL-4 DCs are characterized by a mature and activated phenotype based on surface expression of CD80, CD83, CD86 and CD209.
    ***Optional:*** Stimulation of autologous CD8^+^ T cells can be performed with either fresh or cryopreserved mature IL-4 DCs.

**Figure 3:**
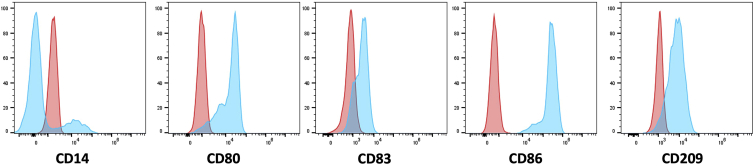
Phenotypic profile of mature IL-4 DCs Representative example of the expression of characteristic markers of mature IL-4 DC (blue) compared to isotype controls (red).

**Figure 4:**
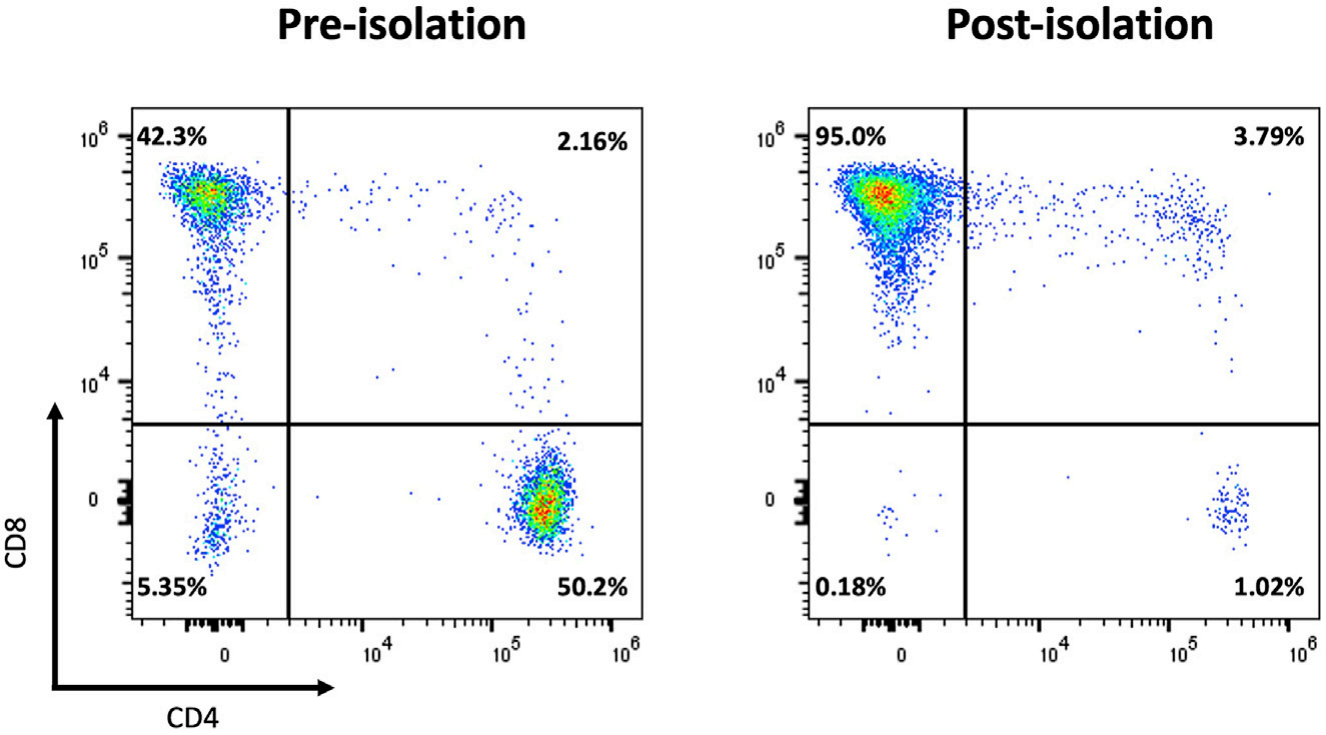
Representative flow cytometric plots of the percentage of CD8^+^ cells before and after immunomagnetic enrichment Gates were set on viable CD3^+^ cells by gating on the forward scatter (FSC) and side scatter (SSC) pseudocolor plots, followed by gating on the CD3^+^ cells. A purity between 90%–99% can be expected after immunomagnetic isolation.
  b. Perform magnetic labeling and separation of CD8^+^ T cells according to the manufacturer’s instructions.
  c. After isolation, check T-cell purity (Figure 4) and viability of the CD8^+^ selected cell fraction by flow cytometry as described in step 7a of the ‘before you begin’ section (problem 2).
    ***Note:*** If viability is <90%, take it into account in further downstream assays.

7. Isolation of CD8^+^ T cells from the CD14^-^ cell fraction.
  ***Note:*** For this step, a similar isolation protocol as described for CD14^+^ monocytes, is used. CD8^+^ T cells are specifically labeled with CD8^+^ magnetic microbeads according to the manufacturer’s instructions: https://www.miltenyibiotec.com/upload/assets/IM0001984.PDF. Prior to the magnetic labeling, the percentage of CD8 T cells is determined.
  a. Determine the percentage of CD8^+^ T cells in the PBL fraction by surface staining.
    i. Transfer 0.1 × 10^6^ cells and transfer into a non-sterile FACS tubes.
    ii. Stain by adding 5 μL of CD3-phycoerythrin (PE), 5 μL of CD4-PerCP, and 5 μL of CD8-FITC.
    iii. Incubate for 15 min at room temperature in the dark.
    iv. Wash once with 2 mL of FACS buffer. Centrifuge at 480 × *g* for 5 min. Discard buffer.
    v. Add 100 μL of FACS buffer and analyze by flow cytometry.
    vi. Evaluate the percentage of CD3^+^CD8^+^ T cells (Figure 4) and calculate the number of PBL and volumes of reagents for magnetic labeling and separation.

8. Cryopreservation of the CD8^+^ T-cell fraction for later use in the first IVS and the CD8 negative cell fraction for the second IVS.
  a. Centrifuge the cell suspension at 480 × *g* for 5 min and discard buffer.
  b. Resuspend cells at a concentration of 1.0 × 10^7^ per 1.5 mL into cold freezing medium and aliquot the cell suspension at 1.5 mL per cryovial.
  c. Freeze the cryovials at −80°C for no less than 4 h and no more than 12 h in a freezing container e.g., Mr. Frosty (or 2-propanol-filled freezing container) before transferring vials to liquid nitrogen for storage.

***Note:*** Once the freezing medium is added to the cells, one must work as quickly as possible, since cells should have limited exposure to the DMSO at room temperature.

## Key resources table

**Table:** 

REAGENT or RESOURCE	SOURCE	IDENTIFIER
**Antibodies**		

Mouse anti-human CD3 - PE - Clone HIT3a (5 μL/10^5^ cells)	BD Biosciences	Cat# 555340 RRID: AB_395746
Mouse anti-human CD3 - PerCP-Cy5.5 - Clone UCHT1 (2.5 μL/2 × 10^6^ cells)	BD Biosciences	Cat# 560835 RRID: AB_2033956
Mouse anti-human CD4 – PerCP – Clone SK3 (5 μL/10^5^ cells)	BD Biosciences	Cat# 345770 RRID: AB_2868798
Mouse anti-human CD8 – FITC – Clone SK1 (5 μL/10^5^ cells)	BD Biosciences	Cat# 345772 RRID: AB_2868800
Mouse anti-human CD8 – Pacific Blue – Clone 3B5 (2.5 μL/2 × 10^6^ cells)	BD Biosciences	Cat# MHCD0828
Mouse anti-human CD14 – FITC – Clone MfP9 (1 μL/10^5^ cells or 5 μL/2 × 10^6^ cells)	BD Biosciences	Cat# 345784 RRID: AB_2868810
Mouse anti-human CD14 - PE - Clone MfP9 (1 μL/10^5^ cells)	BD Biosciences	Cat# 345785 RRID: AB_2868811
Mouse anti-human CD14 - PE-Cy7 - Clone 61D3 (1 μL/10^5^ cells)	Abcam	Cat# ab155360
Mouse anti-human CD19 - FITC - Clone 4G7 (5 μL/2 × 10^6^ cells)	BD Biosciences	Cat# 345776 RRID: AB_2868804
Mouse anti-Human CD80 - PE - Clone L307.4 (1 μL/10^5^ cells)	BD Biosciences	Cat# 557227 RRID: AB_396606
Mouse anti-Human CD83 - FITC - Clone HB15e (1 μL/10^5^ cells)	BioLegend	Cat# 305306 RRID: AB_314514
Mouse anti-Human CD86 - PE-Cy7 - Clone 2331 (FUN-1) (1 μL/10^5^ cells)	BD Biosciences	Cat# 561128 RRID: AB_10563077
Mouse anti-human CD209 - FITC - Clone DCN46 (3 μL/10^5^ cells)	BD Biosciences	Cat# 551264 RRID: AB_394122
Mouse IgG1, kappa Isotype Control - FITC - Clone X40 (1 μL/10^5^ cells)	BD Biosciences	Cat# 345815 RRID: AB_2868833
Mouse IgG1, kappa Isotype Control - PE - Clone X40 (1 μL/10^5^ cells)	BD Biosciences	Cat# 345816 RRID: AB_2868834
Mouse IgG1, kappa Isotype control - PE-Cy7 - Clone 15H6 (1 μL/10^5^ cells)	Abcam	Cat# ab154457
Mouse IgG1, kappa Isotype Control - PE/Cyanine7 - Clone MOPC-21 (1 μL/10^5^ cells)	BioLegend	Cat# 400126 RRID: AB_326448
Mouse IgG2b, kappa Isotype Control - FITC - Clone MPC-11 (1 μL/10^5^ cells)	BD Biosciences	Cat# 559532 RRID: AB_479701
Mouse IgG2b, kappa Isotype Control - PE - Clone 27–35 (1 μL/10^5^ cells)	BD Biosciences	Cat# 556656 RRID: AB_396520
Mouse anti-human HLA-A2 - unconjugated (100 μL/10^6^ cells)	In-house produced with a BB7.2 hybridoma.	N/A
Fluorescein isothiocyanate (FITC)-conjugated rabbit anti-mouse antibody (1 μL/10^6^ cells)	Dako	Cat# F0313
Mouse gamma globulin (1 μL/10^6^ cells)	Jackson Immuno Research Europe Ltd.	Cat# 015-000-002

**Biological samples**		

Unpurified human buffy coat (25–50 mL) (both male and female human subjects, ages range from 18–70)	Healthy volunteers	Flemish Red Cross

**Chemicals, peptides, and recombinant proteins**		

Dulbeco’s PBS without Ca^2+^ and Mg^2+^	Life Technologies	Cat# 14200067
Ficoll-Paque PLUS density gradient media	GE Healthcare	Cat# 17-1440-03
Ethylenediaminetetraacetic acid (EDTA)-Na_2_ salt	Merck	Cat# 1084180250
Sodium azide (NaN_3_), CAS No. 26628-22-8	Merck	Cat# 769320
Ammonium chloride (NH_4_Cl)	Merck	Cat# 101145
Potassium bicarbonate (KHCO_3_)	Merck	Cat# 104854
Bovine serum albumin (BSA)	Sigma Aldrich	Cat# A1662-1L
Human serum albumin (hAB)	Sanbio	Cat# A25761
RPMI 1640 Medium	Life Technologies	Cat# 21875034
Opti-MEM™ I Reduced Serum Medium, no phenol red	Life Technologies	Cat# 11058021
BD FACSFlow	BD Biosciences	Cat# 342003
Dimethyl sulfoxide (DMSO)	Sigma Aldrich	Cat# D2650-100mL
Streptavidin-Allophycocyanin (APC)	ProZyme Europe ApS	Cat# PJ27S
LIVE/DEAD Fixable Aqua Dead Cell Stain Kit, for 405 nm excitation	Life Technologies	Cat# L34957
7-AAD (7-Amino-actinomycin D) Staining Solution	BD Biosciences	Cat# 559925
Propidium Iodide	Life Technologies	Cat# P3566
Recombinant Human Interleukin-4 (rh IL-4)	ImmunoTools GmbH	Cat# 11340045
Recombinant Human Interleukin-7 (rh IL-7)	ImmunoTools GmbH	Cat# 11340073
Recombinant Human Interleukin-15 (rh IL-15)	ImmunoTools GmbH	Cat# 11340155
Recombinant Human Interleukin-21 (rh IL-21)	ImmunoTools GmbH	Cat# 11340213
Recombinant Human Granulocyte Macrophage Colony Stimulating Factor (rhGM-CSF)	ImmunoTools GmbH	Cat# 11343127
Prostaglandin E_2_	Pfizer	CNK0815-027
Human tumor necrosis factor alfa (TNF-α)	Miltenyi	Cat# 130-094-015
WT1_37-45_-specific HLA-A2 monomers	David A. Price	Custom
WT1_37-45_ peptides	JPT Peptide Technologies GmbH	(N-VLDFAPPGA-OH)

**Critical commercial assays**		

Human CD8 MicroBeads	Miltenyi Biotec B.V.	130-045-201
CliniMACS® human CD14 Microbeads	Miltenyi Biotec B.V.	200-070-118

**Software and algorithms**		

FlowJo v.10.8.0	FlowJo LLC	https://www.flowjo.com/
GraphPad Prism 9.3.1	GraphPad Software, LLC	https://www.graphpad.com/scientific-software/prism/
CytExpert 2.3.0.84	Beckman Coulter	https://www.beckman.pt/flow-cytometry/research-flow-cytometers/cytoflex/software

**Other**		

LS column	Miltenyi Biotec	Cat# 130-042-401
QuadroMACS Separator	Miltenyi Biotec	Cat# 130-091-051
MACS MultiStand (magnetic stand)	Miltenyi Biotec	Cat# 130-042-303
Polystyrene FACS tubes	MLS	Cat# A10065N
Sterile FACS tubes for peptide pulsing	VWR International BVBA	Cat# 352054
CELLSTAR® 15 mL Falcon tubes	Greiner Bio-One	Cat# 188271
CELLSTAR® 50 mL Falcon tubes	Greiner Bio-One	Cat# 227261
CELLSTAR® T175 culture flask	Greiner Bio-One	Cat# 660175
CELLSTAR® 24-well plate	Greiner Bio-One	Cat# 662160
2 mL cryovials	Sarstedt	Cat# 72-694-006
ABX Micros ES60 Hematology Analyzer	Horiba	N/A
CytoFLEX Flow Cytometer	Beckman Coulter	N/A
BD FACSAria II	BD Bioscience	N/A
XRAD-320	Precision X-Ray	N/A

## Materials and equipment

**Table:** PBS (1×) buffer

Reagent	Final concentration	Amount
ddH_2_O	N/A	900 mL
PBS (10×)	1×	100 mL
**Total**	**N/A**	**1 L**

Filter sterilize, keep sterile, store at 4°C for up to 3 months.

**Table:** EDTA buffer

Reagent	Final concentration	Amount
ddH_2_O	N/A	1 L
EDTA-Na_2_ salt	100 mM	37.224 g
**Total**	**N/A**	**1 L**

Filter sterilize, keep sterile, store at 4°C for up to 12 months.

**Table:** PBS/EDTA buffer

Reagent	Final concentration	Amount
PBS (1×)	N/A	990 mL
EDTA buffer (100 mM)	1 mM	10 mL
**Total**	**N/A**	**1 L**

Filter sterilize, keep sterile, store at 4°C for up to 3 months.

**Table:** MACS buffer

Reagent	Final concentration	Amount
PBS (1×)	N/A	963.25 mL
EDTA buffer (100 mM)	2 mM	20 mL
Bovine serum albumin (30%)	10 nM	16.75 mL
**Total**	**N/A**	**1 L**

Filter sterilize, keep sterile, store at 4°C for up to 3 months.

**Table:** Sodium azide stock solution (1% w/v)

Reagent	Final concentration	Amount
ddH_2_O	N/A	1 L
Sodium azide (NaN_3_)	1%	10 g
**Total**	**N/A**	**1 L**

Filter sterilize, keep sterile, store at RT for up to 12 months.

**Table:** FACS buffer

Reagent	Final concentration	Amount
BD FACSFlow	N/A	946.7 mL
Bovine serum albumin (30%)	0.1%	3.3 mL
Sodium azide (NaN_3_; 1%)	0.05%	50 mL
**Total**	**N/A**	**1 L**

Store at 4°C for up to 6 months.

**Table:** Lysis buffer (10×) stock solution

Reagent	Final concentration	Amount
ddH_2_O	N/A	1 L
Ammonium chloride (NH_4_Cl)	1,55 M	82.9 g
Potassium bicarbonate (KHCO_3_)	0,1 M	10 g
EDTA-Na_2_ salt	1 mM	370 mg
**Total**	**N/A**	**1 L**

Filter sterilize, keep sterile, store at 4°C for up to 12 months.

**Table:** RBC lysis buffer

Reagent	Final concentration	Amount
ddH_2_O	N/A	45 mL
Lysis buffer (10×) stock solution	1×	5 mL
**Total**	**N/A**	**50 mL**

Filter sterilize, keep sterile, store at 4°C for up to 6 months.

**Table:** Freezing medium

Reagent	Final concentration	Amount
FBS	90%	9 mL
DMSO	10%	1 mL
**Total**	**N/A**	**10 mL**

Keep sterile, store at 4°C for up to 6 months.

**Table:** DC medium

Reagent	Final concentration	Amount
RPMI cell culture medium	N/A	48.75 mL
hAB	2,5%	1.25 mL
**Total**	**N/A**	**50 mL**

Keep sterile, store at 4°C for up to 6 months.

**Table:** T cell medium

Reagent	Final concentration	Amount
RPMI cell culture medium	N/A	45 mL
hAB	10%	5 mL
**Total**	**N/A**	**50 mL**

Keep sterile, store at 4°C for up to 6 months.

**Table:** DC-T cell medium

Reagent	Final concentration	Amount
RPMI cell culture medium	N/A	45 mL
hAB	10%	5 mL
IL-7	5 ng/mL	0,25 mL
IL-15	5 ng/mL	0,25 mL
**Total**	**N/A**	**50 mL**

Keep sterile, store at 4°C for up to 7 days. Do not prepare too much excess medium because cytokines degrade easily in the medium.

## Step-by-step method details

The preparatory steps above describe the isolation and generation of mature IL-4 DCs and CD8^+^ T cells from fresh PBMCs. The following sections describe the further use of these cells. The positively selected CD8^+^ T cells are cryopreserved upon isolation, to be then thawed and specifically activated (on day 1) and expanded in two rounds of IVS; first (on day 1) with fresh or thawed autologous mDCs, second with thawed irradiated autologous PBL, originating from the CD14^-^ and CD8^-^ cell fractions that are cryopreserved to be used upon irradiation for second IVS (on day 8 after 1st IVS).

### First IVS of autologous CD8^+^ T cells with WT1_37-45_-peptide pulsed mature IL-4 DCs on day 1

**Timing: 8 days**

In this part of the protocol, fresh or thawed mature IL-4 DCs are pulsed with WT1 peptide prior to co-culture with thawed autologous primary CD8^+^ T cells in a 1:10 ratio in the presence of IL-21 (Immunotools),^2^ representing the first antigen-specific IVS of autologous CD8^+^ T cells. Every 2–3 days, co-cultures are passaged and supplemented with IL-7 and IL-15 boosting the growth of CD8^+^ T cells, until harvest at day 8.

1. Peptide-pulsing of fresh or thawed mature IL-4 DCs.
  a. In case mature IL-4 DCs are cryopreserved, thaw cells by transferring into warm DC medium pre-warmed at 37°C.
  b. Wash by centrifuging at 480 × *g* for 5 min and resuspend mature IL-4 DCs in serum-free RPMI.
  c. Adjust cell density to 2.0 × 10^6^ cells/mL in serum free RPMI.
  d. Add 10 μg of WT_137-45_ peptide per mL (peptide pulsed condition, PP) to the mature IL-4 DCs and incubate tubes in motion for 1 h on a roller.
    ***Note:*** It is recommended to include a negative control (non-peptide pulsed, NP).
  e. Centrifuge the tubes at 480 × *g* for 5 min, 20°C–22°C, discard supernatant.
  f. Resuspend the cell pellet with 4 mL of T cell medium.
  g. Count viable cells and adjust the concentration to 0.5 × 10^6^ cells/mL.
2. Setting up co-cultures of mature IL-4 DC and autologous CD8^+^ T cells in a 24-well plate.
  a. Thaw cryopreserved autologous CD8^+^ T cells.
  b. Transfer into T cell medium pre-warmed at 37°C.
  c. Wash and resuspend naïve CD8^+^ T cells in T cell medium to a concentration of 5.0 × 10^6^ cells/mL.
  d. Let the cells rest for 1 h at 37°C; then measure viability.
  e. Transfer 1 mL/well of the CD8^+^ T cell suspension (i.e., 5.0 × 10^6^ cells) into a 24-well plate.
    ***Note:*** Include wells for the appropriate controls. Initially, 1 well/condition is prepared.
  f. Transfer 1 mL of the PP DC suspension (i.e., 0.5 × 10^6^ cells/mL) to each well containing autologous CD8^+^ T cells. Use NP DCs for the control condition.
    ***Note:*** Minimal required conditions include NP DCs and PP DCs with CD8^+^ T cells in wells of a 24-well plate at a DC:T cell ratio of 1:10.
  g. Add 30 ng/mL IL-21 to the co-cultures.
  h. Incubate the co-cultures at 37°C, 5% CO_2_ for 8 days.
    ***Note:*** Observe co-cultures daily for changes in color of media and cell confluence. Each donor responds differently, therefore, cells should be split based on their confluence and media color. A typical culture needs to be passaged every 2–3 days until harvest at day 8. Typically, one single well will be expanded to eight wells after activation with mature IL-4 DCs within 8 days. If necessary, the IVS round could be extended to 10 days (problem 4).

3. Passaging T cells and addition of IL-7 and IL-15.
  a. When cells are confluent, resuspend by pipetting and transfer 1 mL into an empty well of a 24-well plate and fill up each well up to 2 mL with DC:T cell medium.
  b. If cells are not yet confluent, refresh DC:T cell medium by carefully removing 1 mL of medium and adding 1 mL of fresh DC:T cell medium.
  c. Incubate the cells at 37°C with 5% CO_2_.

### Determine amount of antigen-specific T cells after 1^st^ IVS on day 8

**Timing: 3 h**

***Optional:*** This part of the protocol analyzes the amount of antigen-specific CD8^+^ T cells 8 days after the initiation of the first IVS using epitope-specific-TCR-binding MHC-I tetramers by flow cytometry. This provides an indication, but no guarantee yet, of the success of the expansion. After the first IVS, the percentage of WT1_37-45_-specific CD8^+^ T cells is expected to be 0.27 ± 0.21% (problem 5). Hence, highly likely a second IVS is needed to expand antigen-specific CD8^+^ T cells to sufficient numbers for downstream analyses.

4. Harvest T cells from IVS1.
  a. Resuspend the 1^st^ IVS co-cultures by firmly pipetting and transfer to a 50 mL Falcon tube.
  b. Wash wells by adding 2 mL of PBS-EDTA buffer in order to collect remaining cells.
  c. Check under the microscope if you harvested all cells. If not, repeat step 4b.
  d. Centrifuge at 480 × *g* for 5 min, decant wash buffer.
  e. Resuspend cell pellet in fresh T cell medium supplemented with another 10% of hAB serum for a final concentration of hAB serum of 20%.
    ***Note:*** The concentration hAB serum in the T cell medium is now 20%. Hence the final concentration hAB serum in the co-culture when adding the feeder cells should be 10% again.
  f. Count cells and assess cell viability.
  g. Bring cells to a concentration of 5.0 × 10^6^ cells/mL in T cell medium.
5. HLA-A2/WT1_37-45_ tetramer staining.
  a. Transfer 2.0 × 10^6^ of the CD8^+^ T cells to a non-sterile FACS tube.
  b. Add 2 mL of FACS buffer and wash by centrifuging for 5 min at 480 × *g* and carefully discard buffer.
  c. Centrifuge HLA-A2/WT1_37-45_ tetramers at 17,000 × *g* for 5 min to spin down any potential aggregates.
  d. Add 4 μL of HLA-A2/WT1_37-45_ tetramers for cell staining and incubate for 15 min at 37°C.
  e. Add 2 mL of FACS buffer and centrifuge at 480 × *g* for 5 min.
  f. Remove supernatant and stain cell pellet in the remaining FACS buffer volume with the fluorescently labeled antibody panel C (Table 2) for 30 min at 4°C.

**Table 2:** Antibody staining panel C for flow cytometric analysis to define the frequency of HLA-A2/WT1_37-45_ tetramer^+^ T cells after 1^st^ IVS

Panel C		
Target	Fluorophore	Volume added
CD3	PerCP-Cy5.5	2.5 μL
CD8	Pacific Blue	2.5 μL
CD14	FITC (dump channel)	5 μL
CD19	FITC (dump channel)	5 μL
Viability dye	Aqua	1 μL
  g. Add 2 mL of FACS buffer and wash by centrifuging for 5 min at 480 × *g* and carefully discard buffer.
  h. Resuspend cell pellet in 500 μL of FACS buffer.
  i. Analyze samples on a FACSAria II flow cytometer. Figure 3 gives an example of the expected HLA-A2/WT1_37-45_ tetramer staining after 1^st^ IVS (problem 3).

***Note:*** In case antigen-specific T cells are sorted after the 1^st^ IVS for downstream analysis, it is advisable to perform the flow cytometric analysis on the same device as in this case only one antibody panel is needed.

### Second IVS of CD8^+^ T cells with WT1_37-45_ peptide-pulsed autologous CD14^-^ CD8^-^ PBL on day 8

**Timing: 8 days**

In the following steps, autologous CD14^-^ CD8^-^ PBL, which serve as the feeder cells for the CD8^+^ T cells after 1^st^ IVS, are thawed and thereafter irradiated to prevent further cell division. Next, irradiated PBLs are WT1_37-45_ peptide pulsed and cultured with autologous 8-day WT1-loaded DC:CD8 T cell co-cultures in the presence of IL-21. Every 2–3 days, these co-cultures are passaged and supplemented with IL-7 and IL-15, until harvest at day 16 (problem 4). On day 16, expanded co-cultures are analyzed for the presence of WT1-specific CD8^+^ T cells by HLA-A2/WT1_37-45_ tetramers.

6. Thaw cryopreserved PBL as described in step 2a and adjust the cell density to 2.5 × 10^6^ cells/mL using T cell medium in a 50 mL Falcon tube.
7. Irradiate the autologous PBL with 35 Gy using an XRAD-320 device.

**CRITICAL:** One must follow an irradiation safety training before operating an XRAD-320 device.

8. PBL peptide pulsing.
  a. Seed 5.0 × 10^6^ of irradiated cells in a 24-well plate and incubate at 37°C for 1 h.
  b. Carefully aspirate the medium, removing non-adherent cells.
  c. Add 10 μg of WT1_37-45_ peptide in 1 mL of serum-free RPMI to the adherent cells.
  d. Incubate on a shaker in slow shaking motion at 37°C for 1 h.
9. Restimulation of autologous T cells.
  a. Add 5.0 × 10^6^ CD8^+^ T cells of step 4 to the corresponding well in the presence of 30 ng of IL-21 per mL of media.
  b. Incubate the cells at 37°C with 5% CO_2_ for 7 days.
  c. Passage co-cultures every 2–3 days as described in step 3.
  d. On day 16, after 8 days of expansion, harvest T cells and stain with HLA-A2/WT1_37-45_ tetramers as previously described in step 5 to analyze the presence of WT1_37-45_-specific CD8^+^ T cells (problem 6).

## Expected outcomes

**Figure 5:**
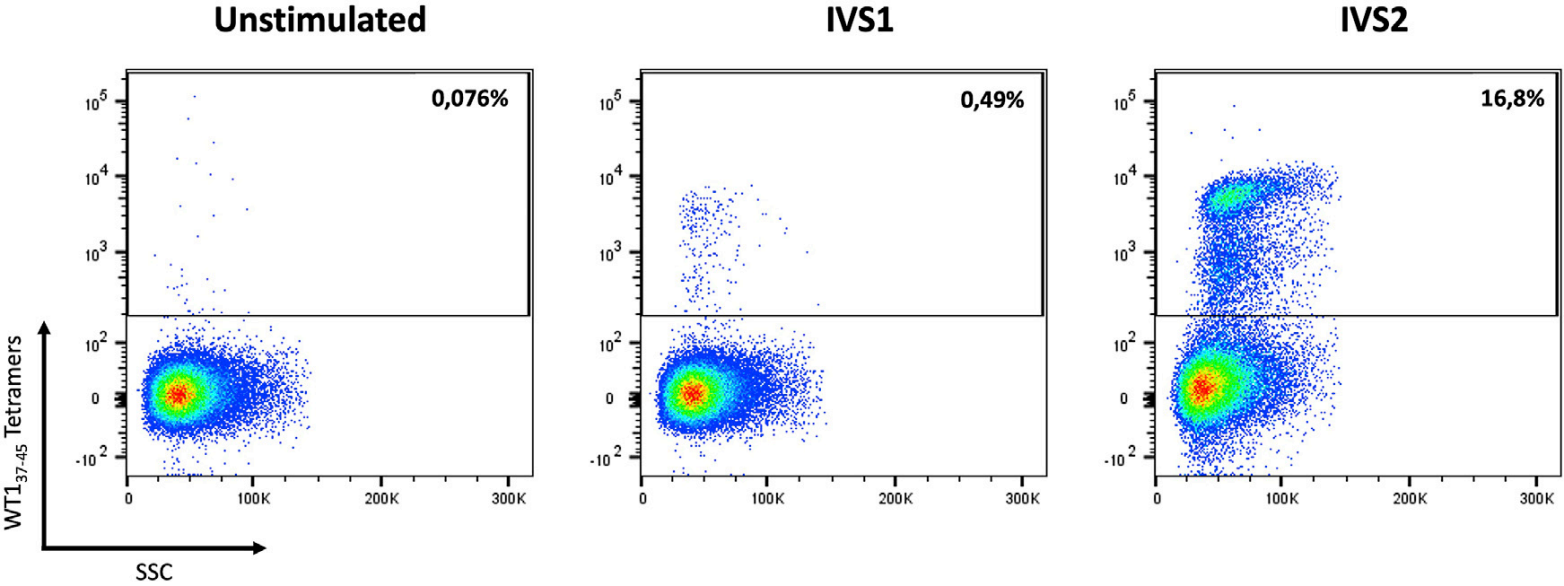
Representative example of WT1_37-45_-specific T cells after one or two rounds of *in vitro* stimulations (IVS) CD8^+^ T cells were, first, stimulated with autologous unpulsed (unstimulated) or WT1_37-45_ peptide-pulsed (IVS1) mature IL-4 DCs and, second, with autologous WT1_37-45_ peptide-pulsed CD14^-^ CD8^-^ PBL (IVS2). Samples were stained with HLA-A2/WT1_37-45_-specific tetramers.

The described WT1_37-45_-specific T-cell expansion protocol is developed to generate T-cell clones specific for the TAA WT1_37-45_ epitope. As illustrated by the exemplary flow cytometric results (Figure 5), after the 2^nd^ IVS, WT1_37-45_-specific T cells expanded significantly in comparison to the 1^st^ IVS and unstimulated T cells. On average, an increase in the frequency of WT1_37-45_ tetramer^+^ T cells can be expected after the 2^nd^ IVS. The generated WT1_37-45_-specific T-cell clones can serve as starting material for downstream assays to study T-cell characteristics. T-cell clones can be used to analyze the antigen-specific TCR repertoire of an individual, to build algorithms for the evaluation of the immune signature or for the training of prediction models for targeted therapy. Another application for the generated T-cell clones can be single-cell sequencing for the isolation of WT1_37-45_-specific TCRs to develop antigen-specific TCR-engineered adoptive T-cell therapies. In addition to analysis of T-cell clones, this protocol can also serve as a tool to evaluate antigen-presenting capacity of an antigen-presenting cell of interest.

## Limitations

For the expansion of antigen-specific CD8 T cells, T cells with TCRs that can recognize the epitope of interest should be present at sufficient frequencies in the collected blood sample to interact with the antigen-presenting DCs. Accordingly, the antigen-specific TCR repertoire variability between donors, some lacking WT1-specific T-cell clones, may limit the expansion of antigen-specific T cells. Hence, one should screen multiple donors before concluding on the immunogenicity of a given antigen or epitope. Although this protocol focused on the expansion of T-cell clones reactive to HLA-A2-restricted WT1_37-45_ peptide, this protocol can be used for other WT1 peptides, but also other TAAs and HLA types. Moreover, the use of full-length WT1 messenger RNA (mRNA) transfected DCs instead of WT1_37-45_ peptide-pulsed DCs would result in full epitope repertoire presentation. This alternative strategy using mRNA transfection would increase the diversity of epitope-specific T-cell clones expanded with this protocol targeting the same TAA. However, the exact epitopes that expanded T cells are reacting against may prove difficult to discern as commercial HLA/peptide multimers are only available for a limited number of epitopes. Furthermore, it should be noted that tetramers only provide information on the frequency of antigen-specific T cells and provide little information about T-cell potency, functionality, or avidity (no clear correlation between the percentage of tetramer^+^ T cells and T-cell activity). Therefore, functional analysis is needed when expanded antigen-specific T cells are intended for adoptive T-cell therapies. Lastly, other autologous antigen-presenting cells can be used for the expansion of antigen-specific T cells, e.g., IL-15 DCs.^1^ However, protocol optimization will likely be needed when using different antigen-presenting cells.

## Troubleshooting

### Problem 1

Low yield of mature IL-4 DCs (cfr step 5 of the ‘before you begin’ section).

### Potential solution

DCs attach firmly to the plastic surface of the cell culture flask. One could insufficiently harvest the maturated IL-4 DCs. Hence always check by microscopy that all DCs have been collected after cell harvesting. In case that DCs are still adherent to the surface, add PBS-EDTA buffer and incubate at 4°C. After a 10-min incubation period, firmly tap on the back of the flask to detach the cells for harvest.

### Problem 2

Low purity of T cells (cfr step 7 of the ‘before you begin’ section).

### Potential solution

An incorrect amount of magnetic beads could have been used or the microbeads may have not been vortexed before being added to the cell suspension. Vortex the beads before use and mix by pipetting with the cell suspension. As described in the Protocol, check purity of isolated naïve CD8^+^ T cells before start of the co-culture.

### Problem 3

No WT1-tetramer^+^ T-cell populations (step 5).

### Potential solution

This could be due to unsuccessful staining of T cells caused by poor quality of the tetramers prepared. After tetramerization, perform a quality control check by using the tetramers against a T-cell line or a clone of known TCR specificity.

### Problem 4

Antigen-specific CD8^+^ T cells do not expand (step 5).

### Potential solution

After IVS, T-cell exhaustion or activation-induced cell death could be triggered. Restimulate T-cell clones with fresh feeder cells (cfr step 6 onwards).

### Problem 5

Expansion of CD8^-^ T cells during the culture (step 5).

### Potential solution

CD8^+^ T-cell purity after MACS isolation is expected to be >95%. However, if CD8^-^ T cells are present after isolation, these could expand during IVS, decreasing the purity and yield of antigen-specific CD8^+^ T cells. Always check the purity of the CD8^+^ T cells after isolation.

### Problem 6

Feeder outgrowth (step 7).

### Potential solution

This could be caused by insufficient irradiation. Use the recommended or a superior dose for irradiating the PBL.

## Resource availability

### Lead contact

Further information and requests for resources and reagents should be directed to and will be fulfilled by the lead contact, Eva Lion (Eva.Lion@uantwerpen.be).

### Materials availability

This study did not generate new unique reagents.

## Data Availability

This protocol did not generate data.

## References

[1] Anguille S., Smits E.L.J.M., Cools N., Goossens H., Berneman Z.N., Van Tendeloo V.F.I. (2009). Short-term cultured, interleukin-15 differentiated dendritic cells have potent immunostimulatory properties. J. Transl. Med..

[2] Chapuis A.G., Ragnarsson G.B., Nguyen H.N., Chaney C.N., Pufnock J.S., Schmitt T.M., Duerkopp N., Roberts I.M., Pogosov G.L., Ho W.Y. (2013). Transferred WT1-reactive CD8+ T cells can mediate antileukemic activity and persist in post-transplant patients. Sci. Transl. Med..

